# Complete genome sequences of *Mycobacterium smegmatis* phages Ashballer and Bombitas

**DOI:** 10.1128/mra.00990-23

**Published:** 2024-01-17

**Authors:** Kristi M. Westover, Alexis R. Atkinson, Abby G. Bowers, Amaya L. Brown, James C. Ferebee, Chase A. Keisler, Kaylyn L. Lee, Amaya O. Payton, Lidia A. Peralta, Julianne V. Phu, Khamryn A. Pollock, Maya G. Scott, Bryson E. Vaughan, Karissa M. Wilczak, Victoria J. Frost

**Affiliations:** 1 Department of Biology, Winthrop University, Rock Hill, South Carolina, USA; Portland State University, Oregon, USA

**Keywords:** mycobacteriophage

## Abstract

We report the discovery of two mycobacteriophages isolated from soil in Rock Hill, South Carolina. Ashballer has a genome sequence length of 52,231 bp, while Bombitas is relatively larger at 110,129 bp. Both have siphovirus morphologies and have temperate lifecycles.

## ANNOUNCEMENT

**Fig 1:**
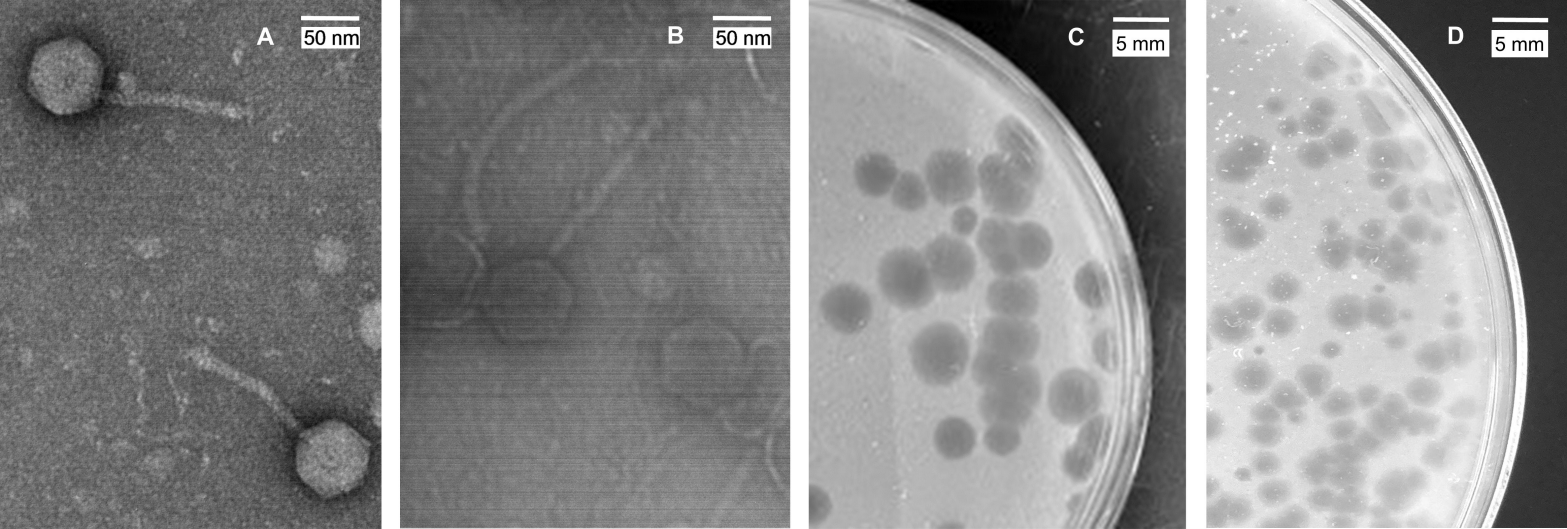
Transmission electron micrographs of mycobacterium phages Ashballer (**A**) and Bombitas (**B**). Phage lysates were negatively stained with 1% uranyl acetate, and images were taken with JEOL JEM-1230 TEM at 100 kV acceleration voltage. Ashballer (**C**) and Bombitas (**D**) form plaques with a clear center surrounded by a turbid edge.

With the recent compassionate use of bacteriophages to treat antibiotic-resistant bacterial infections, interest in phage therapy and associated clinical trials is increasing (1, 2). Consequently, the need for large, diverse arsenals of phage stocks is crucial. Here, two mycobacteriophages found in damp soil near tree roots on the campus of Winthrop University, Rock Hill, SC (see Table 1 for GPS coordinates), were isolated on bacterial host *Mycobacterium smegmatis* mc^2^155. Soil samples were washed (2 hours) using 7H9 broth containing 1 mM CaCl_2_, centrifuged (4,000 rpm) for 10 minutes using standard procedures (https://seaphagesphagediscoveryguide.helpdocsonline.com/home), and the supernatant was filtered (0.22 µm). Mycobacteriophages were enriched by inoculating the soil filtrates with *M. smegmatis* and agitating (250 rpm) at 37°C for 5 days. The enriched culture was re-filtered, plated in soft agar with *M. smegmatis* on 7H9 agar plates, and incubated at 37°C. Both Ashballer and Bombitas created plaques with clear centers and turbid edges. Ashballer had larger plaques (3–5 mm in diameter) compared to Bombitas (≤1.5 mm) (Fig. 1). Transmission electron microscopy revealed that both phages have siphovirus morphologies (Fig. 1).

**TABLE 1:** Phage discovery locations, cluster assignment, and genome assembly results

Phage name	Location site (GPS)	Average coverage (X)	Reads (K)	Cluster	Genome size (bp)	Genome ends	GC content (%)	No. of genes
Ashballer	34.93722 N, 81.03086 W	1,215	447	A1	52,231	3′ single-stranded overhang (5′- CGGATGGTAA-3′)	63.8	95
Bombitas	34.941325 N, 81.034067 W	327	255	J	110,129	3′ single-stranded overhang (5′- ATCC-3′)	60.7	226

Phage DNA was extracted from lysate using the Wizard DNA cleanup kit (Promega), and libraries were constructed using the NEBNext Ultra II FS DNA library prep kit before sequencing with the Illumina MiSeq v3 platform. The resulting 150 bp single-end raw reads were assembled using Newbler v2.9 and checked for accuracy, coverage, and genomic termini using Consed v29 as previously described (3, 4). Sequencing results and phage genome characteristics are listed in Table 1 which includes the predicted number of genes, and phage cluster designation based on gene content similarity (GCS) of at least 35% to phages within the Actinobacteriophage database (https://phagesdb.org/) using the GCS tool at phagesDB and previously described criteria (5, 6).

Genome sequences were annotated using DNA Master V5.23.6 (7) embedded with Glimmer v3.02 (8) and GeneMark v2.5 (9), Starterator (7), Phamerator v3 (10), and BLASTp v2.13.0 (11). Databases PDBmmCIF70, Pfam-A, and NCBI Conserved Domain were selected in HHpred v3 (12) during homology searches. Transfer RNAs were identified using Aragorn v1.1 integrated into DNA Master (7), Aragorn v1.2.38 (13), and tRNAscan-SE v2.0.6 (14). Putative transmembrane domains were assessed using TMHMM v2 (15, 16), SOSUI v1.11 (17), and TOPCONS v2 (18). Default parameters were used for all software tools.

Annotation assigned putative gene functions to approximately 26% of Bombitas’s genome and 40% of Ashballer’s genome. The majority of these functional genes were located in the first 44,000 bps of Bombitas (110,129 bps total) and the first 32,000 bps of Ashballer (52,231 bps total) and transcribed in the rightward direction. These include putative portal proteins, tail proteins, capsid proteins, and lysin proteins. The genomes also contained putative immunity repressors and integrases, consistent with the predicted temperate lifestyle of A1 cluster phages. Overall, both genomes had fewer leftward-transcribed genes, the majority of which had no predicted function and were located in the second half of the genome. As is typical of phages in the J cluster, Bombitas has a number of putative glycosyltransferase genes, concentrated in the first 16,000 bps of the genome.

## Data Availability

The complete genome sequences of phages Ashballer and Bombitas are available in GenBank (accession no. OR195043 and OR195048, respectively). The raw sequencing reads are available in the NCBI SRA under accession no. SRX20165782 and SRX20165786 respectively. The Actinobacteriophage sequencing BioProject accession number is PRJNA488469.
